# Favipiravir as a potent inhibitor of Newcastle disease virus: *in ovo* efficacy, dose-dependent toxicity, and molecular insights into RNA polymerase inhibition

**DOI:** 10.14202/vetworld.2025.2785-2797

**Published:** 2025-09-23

**Authors:** Naeem Aziz Soomro, Zaheer Ahmed Nizamani, Mansoor Tariq, Nazeer Hussain Kalhoro, Mamona Mushtaq

**Affiliations:** 1 Department of Veterinary Pathology, Faculty of Animal Husbandry and Veterinary Sciences, Sindh Agriculture University, Tandojam, Pakistan; 2 Department of Livestock and Fisheries, Sindh Institute of Animal Health, Karachi, Government of Sindh, Pakistan; 3 Dr. Panjwani Center for Molecular Medicine and Drug Research, International Center for Chemical and Biological Sciences, University of Karachi, Karachi 75270, Pakistan

**Keywords:** antiviral therapy, favipiravir, *in ovo* model, Newcastle disease virus, poultry health, RNA-dependent RNA polymerase

## Abstract

**Background and Aim:**

Newcastle disease (ND), caused by velogenic viscerotropic ND virus (VVNDV), remains a major threat to global poultry production, with outbreaks persisting even in vaccinated flocks. No approved antiviral therapy exists for ND, highlighting the urgent need for effective interventions. Favipiravir, a broad-spectrum RNA polymerase inhibitor, has shown promise against several RNA viruses. This study evaluated the toxicity, antiviral efficacy, and molecular mechanisms of favipiravir against VVNDV in an *in ovo* model.

**Materials and Methods:**

Specific pathogen-free embryonated chicken eggs (9–10 days old) were inoculated with VVNDV and treated with graded doses of favipiravir (75–2280 mg/kg/egg biomass). Toxicity was assessed through embryo survival, relative weight, morphological scoring, biochemical markers, and histopathology of liver tissues. Antiviral efficacy was evaluated through embryo survival, growth, hemagglutination (HA) titers, and 50% egg infectious dose (EID_50_). Molecular docking was performed to characterize favipiravir’s interaction with viral RNA-dependent RNA polymerase (RdRp). Statistical analyses included Kruskal–Wallis, analysis of variance, and correlation tests.

**Results:**

Favipiravir displayed dose-dependent toxicity, with the highest dose (2,280 mg/kg) significantly reducing embryo survival (p = 0.027) and inducing hepatic necrosis and elevated alkaline phosphatase and urea levels. In contrast, therapeutic doses of 300 and 600 mg/kg achieved 100% embryo survival, significant weight gains, and complete viral suppression, with undetectable HA activity and EID_50_ values. Favipiravir demonstrated antiviral efficacy by suppressing viral replication and conferring protection against VVNDV. Docking analysis revealed a strong binding affinity of favipiravir to RdRp, primarily mediated by electrostatic interactions and hydrogen bonding with residues Arg1189, Tyr1192, and Ser1288, suggesting inhibition of viral RNA synthesis.

**Conclusion:**

This study provides the first *in ovo* evidence of favipiravir’s efficacy against VVNDV, demonstrating complete viral inhibition at optimized doses while emphasizing the importance of dose-dependent toxicity monitoring. These findings establish favipiravir as a promising antiviral candidate for ND virus control and potentially other RNA viruses of veterinary and One Health importance. Further *in vivo* and field-based studies are warranted to validate its safety, optimize dosing regimens, and evaluate large-scale applicability in poultry production.

## INTRODUCTION

Newcastle disease (ND) is a panzootic viral disease caused by the ND virus (NDV), classified under the genus *Avulavirus* in the family *Paramyxoviridae*. It is an enveloped virus containing a single-stranded, negative-sense, non-segmented RNA genome [1]. NDV is highly contagious and clinically fatal in chickens, making it one of the diseases listed by the World Organization for Animal Health.

ND is a major challenge to the global poultry industry due to its high flock mortality rate of up to 90%, substantial declines in production, and strict trade restrictions imposed on outbreak-affected regions [2]. To date, ND has caused four pandemics, each linked to distinct genotypes and atypical symptoms. Future viral genome variations are possible, and emerging mutations in NDV would likely require the development of new, strain-specific vaccines [3].

Approximately 75% of infectious diseases are zoonotic in nature [4], with animals serving as significant reservoirs and progenitors of novel viruses. In recent decades, human infections caused by severe acute respiratory syndrome coronavirus 1 and 2, *Zika virus*, and Paramyxoviruses such as Hendra, Nipah, and Menangle have underscored the high genetic variability of these pathogens. This variability complicates predictions of viral emergence or re-emergence and creates barriers to rapid vaccine development [5].

In Pakistan, NDV outbreaks have been reported even in vaccinated flocks. Novel viral strains belonging to distinct classes with little to no genetic homology to previously reported isolates caused substantial losses, estimated at approximately USD 200 million in 2012 [6]. At a global scale, the economic burden of ND was estimated at USD 1.4 billion in 2021, accounting for both production losses and control measures [7]. Despite widespread vaccination, outbreaks persist largely because of novel NDV strains with limited genomic similarity to existing vaccine strains, resulting in vaccine failure and continued financial losses [8].

Given these challenges, effective antiviral therapeutics are urgently required to complement vaccination strategies. Favipiravir, a selective inhibitor of RNA-dependent RNA polymerase (RdRp), is a promising candidate. By inducing antiviral mutagenesis, favipiravir generates non-infectious viral progeny incapable of further transmission [9, 10]. Because RdRp is a highly conserved enzyme across RNA viruses, favipiravir demonstrates broad-spectrum antiviral activity [11]. Initially approved for influenza virus infections, it is now under investigation for efficacy against multiple re-emerging RNA viruses [12, 13].

Although vaccination remains the primary strategy for controlling ND, the emergence of novel NDV strains with limited genomic homology to existing vaccinal strains continues to result in vaccine failure and recurring outbreaks. Current commercial vaccines often fail to provide cross-protection, and despite substantial economic losses, no antiviral drugs have been approved for therapeutic use in poultry. Previous *in vitro* and *in vivo* studies have evaluated favipiravir against several RNA viruses, including influenza, Ebola, and Lassa viruses, demonstrating broad-spectrum antiviral activity. However, its application against NDV remains largely unexplored. Importantly, no prior study has comprehensively evaluated favipiravir’s toxicological safety in embryonated chicken eggs, nor has its antiviral efficacy against velogenic viscerotropic NDV (VVNDV) been validated in an *in ovo* model. Furthermore, mechanistic insights into how favipiravir interacts with the RdRp of NDV have not been fully elucidated, leaving a critical knowledge gap in both applied veterinary virology and antiviral drug discovery.

The present study was designed to bridge this gap by systematically assessing the toxicity profile and antiviral efficacy of favipiravir against VVNDV using an *in ovo* chicken embryo model. Specifically, the study aimed to:

1. Determine the dose-dependent toxicity of favipiravir in embryonated chicken eggs through survival analysis, weight measurements, morphological evaluation, biochemical profiling, and histopathology.
2. Evaluate the therapeutic efficacy of favipiravir against VVNDV by monitoring embryo survival, embryo growth, viral titers (hemagglutination [HA] assay and 50% egg infective dose [EID_50_]), and morphological protection.
3. Perform molecular docking studies to elucidate the interaction patterns of favipiravir with NDV RdRp, providing mechanistic evidence for its antiviral activity.

By integrating *in ovo* experimentation with *in silico* docking analysis, this study provides the first experimental evidence of favipiravir’s potential as an antiviral candidate against NDV, while also laying the groundwork for its broader application against RNA viruses of veterinary and One Health significance.

## MATERIALS AND METHODS

### Ethical approval

All experimental procedures followed institutional guidelines for the use of embryonated eggs and were approved by the Ethical Committee with approval number 153 BASAR-11, Faculty of Animal Husbandry and Veterinary Sciences, Sindh Agriculture University, Tandojam.

### Study period and location

The study was conducted for the period of 8 months (15 September 2023 to 15 May 2024), at Sindh Institute of Animal Health (SIAH).

### Study design and experimental groups

Favipiravir was evaluated for its antiviral potential against VVNDV in an *in ovo* model. As no literature documents favipiravir dosing in embryonated chicken eggs, a toxicity assay was performed across a dose range extrapolated from mammalian studies.

Embryos were randomly assigned to control and treatment groups using a random number generator. All outcome assessors were blinded during morphological scoring and histopathological examination. To ensure reproducibility, each experiment was conducted in triplicate across independent batches of specific pathogen-free (SPF) embryonated eggs. Nine-to ten-day-old SPF eggs were obtained from the Sindh Institute of Animal Health (SIAH), incubated at 37°C with 55% relative humidity, and monitored by candling every 12 h. Nonviable embryos were collected for further analysis. The trial period lasted 10 days.

### Preparation of favipiravir and viral inoculum

#### Favipiravir preparation

Favipiravir (Piravir, Getz Pharma) was dissolved in distilled water at 50°C to yield a stock solution of 11 mg/mL (70.01 mM) and stored at −80°C, following datasheet recommendations [14].

#### Viral inoculum preparation

A genotype VII VVNDV strain, passage 3, was obtained from SIAH to maintain genetic stability and virulence. Viral titers were calculated as EID_50_ using the Reed and Muench method [15]. Antibiotics (penicillin, streptomycin, and gentamicin) were added to the inoculum.

### *In ovo* toxicity assay

Favipiravir doses of 200, 541, 1,140, and 2,280 mg/kg were administered through the allantoic route to evaluate toxicity. Dose selection was based on mammalian data and human therapeutic ranges. Biomass was calculated relative to egg weight: At day 10 of incubation, embryos represent approximately 9% of the egg’s original weight (e.g., 4.5 g for a 50 g egg) [16].

Each group included 10 eggs (10 days old). Controls included an untreated negative group and a sham group injected with sterile water.

### Assessment of embryo survival, growth, and morphology

- Survival monitoring: Viability was assessed every 12 h by candling (movement and intact vasculature)
- Relative embryo weight: Embryos were weighed before and after inoculation, and weight-to-egg weight percentages were calculated
- Morphological scoring: Embryos and annexes were inspected for circulatory changes, malformations, and organ abnormalities. Lesions were scored: no sign (0), mild (1), moderate (2), severe (3).

### Biochemical analysis of amnio-allantoic fluid (AAF)

AAF was collected from embryos that died between days 13 and 16 post-incubation and survivors on day 17. Samples were analyzed using a biochemical analyzer (Mindray BS-200, Mindray Bio-Medical Electronics Co., Ltd. Shenzhen, China) and stored at −80°C.

Parameters included:

- Kidney function: Creatinine and uric acid
- Liver function: Alanine transaminase (ALT), aspartate aminotransferase (AST), alkaline phosphatase (ALP)
- Bone marker: ALP
- Inflammation: C-reactive protein.

Normal ranges were derived from controls due to limited reference data for embryonic physiology.

### Histopathological examination

- Sample collection: Livers from dead embryos and controls were fixed in 10% formaldehyde
- Processing: Tissues underwent graded ethanol dehydration, xylene clearing, and paraffin embedding
- Sectioning and staining: 5 μm paraffin sections were stained with hematoxylin and eosin using an automated stainer (Labtron ASS-30LS, USA).
- Mounting: Distyrene-Plasticizer-Xylene Mounting medium (DPX), Bio Rays Pakistan) was applied, and sections were air-dried before microscopic evaluation
- Microscopy and scoring: Sections were examined at 10× magnification (Nikon E-100 microscope, Japan, 25 μm resolution). Lesions were scored:
  - 0 = No lesions
  - 1 = Mild (focal inflammation/minimal infiltration)
  - 2 = Moderate (localized inflammation, degeneration, mild structural disruption)
  - 3 = Severe (widespread damage, necrosis, significant infiltration)
  - 4 = Diffuse severe lesions (extensive necrosis, fibrosis, widespread infiltrates).

Two independent pathologists performed blinded evaluations.

### Evaluation of antiviral efficacy

VVNDV (0.1 mL) was inoculated through the chorioallantoic route. Favipiravir (75, 150, 300, and 600 mg/kg/egg biomass) was injected into the albumen at the pointed end. Survival and embryo weights were monitored every 12 h.

- Virology assays: AAF was collected at 24, 48, and 72 h post-inoculation for HA titers and EID_50_ using the method described by Reed and Muench [15]. Mean death time was also calculated as described by Reed and Muench [15]. Viral replication was assessed by HA activity and EID_50_ values.

### Molecular docking analysis (MDA)

#### Docking procedure

Docking was performed using the molecular operating environment (MOE) suite version 2019.01 (Chemical Computing Group, Montreal, Canada). Blind docking explored the entire RdRp protein surface. The Alpha Triangle algorithm generated conformations, scored by London dG and refined using Generalized Born Volume Integral/Weighted Surface Area ΔG. Out of 500 initial conformations, 200 were retained, and the densest binding cluster was selected. Induced-fit docking was performed, generating 30 poses; the top 5 were analyzed. Visualizations were performed with University of California, San Francisco (UCSF) Chimera and ChimeraX(Resource for Biocomputing, Visualization, and Informatics at UCSF, USA).

#### Interaction analysis

Protein-ligand interactions were characterized using protein ligand interaction profiler and MOE modules [17]. Hydrogen bonds, electrostatic, and non-electrostatic contacts were identified, providing mechanistic insight into RdRp inhibition.

### Statistical analysis

Data were analyzed using Statistical Package for the Social Sciences v27 (IBM Corp., NY, USA).

- Normality testing: Shapiro–Wilk
- Non-parametric tests: Kruskal–Wallis with Dunn’s *post hoc* (Benjamini–Hochberg false discovery ratecorrection). Effect size calculated as ε^2^
- Parametric tests: One-way analysis of variance (ANOVA) with Tukey’s honestly significant difference for normally distributed biochemical markers
- Significance: p ≤ 0.05. Data expressed as mean ± standard deviation or median (interquartile range).

## RESULTS

### Toxicity assessment of favipiravir *in ovo*

#### Embryo survival time

Favipiravir treatment affected embryo survival in a dose-dependent manner (Table 1). Low-to-moderate doses (Groups B, C, and D: 200, 540, and 1,140 mg/kg, respectively) did not significantly affect survival compared with the control. However, the highest dose (Group E: 2,280 mg/kg) significantly reduced survival (p = 0.027), confirmed by Dunn’s *post hoc* test. The effect size (ε^2^ = 0.23) indicated a large magnitude of effect, highlighting dose-limiting toxicity at higher concentrations.

**Table 1:** Embryo survival time after exposure to variable doses of favipiravir.

Group (dose)	Embryo survival time (days) median (IQR)	Kruskal-Wallis p-value	Dunn’s *post hoc*	

			Significant versus control	p-value (FDR adjusted)
A (control)	17 (17–17)	0.027*	-	-
B (200 mg/kg)	17 (17–17)		NS	0.675
C (540 mg/kg)	17 (14–17)		NS	0.496
D (1,140 mg/kg)	17 (14–17)		NS	0.372
E (2,280 mg/kg)	13 (12–17)		S	0.032*

p-values with asterisks denote statistically significant differences. IQR = Interquartile range, FDR = False discovery rate

#### Relative embryo weight

A dose-dependent decline in embryo weight relative to egg weight was observed (Table 2). Group E showed reduced median embryo weight; however, the decrease was not statistically significant. The effect size (ε^2^ = 0.149) indicated a small-to-moderate difference between groups, suggesting a downward trend without robust statistical support.

**Table 2:** Embryo weight relative to egg weight (%).

Group (dose)	Median (IQR)	Kruskal-Wallis p-value
A (control)	24.13 (22.94–26.01)	0.075
B (200 mg/kg)	23.50 (22.16–27.20)	
C (540 mg/kg)	24.06 (9.97–25.88)	
D (1,140 mg/kg)	22.73 (8.9–25.24)	
E (2,280 mg/kg)	9.15 (3.80–18.82)	

IQR = Interquartile range

#### Morphological changes

**Figure 1:**
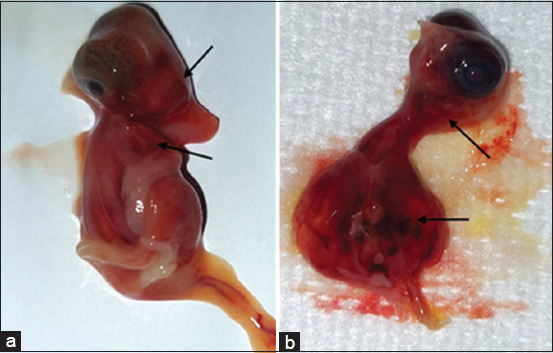
Gross pathological changes due to favipiravir toxicity. (a) Mild hemorrhages (angioedema) observed on the head and thoracic region, corresponding to a hemorrhage score of 1 and (b) Moderate hemorrhages affecting approximately 50% of the body, with visible swelling and discoloration, corresponding to a hemorrhage score of 2, as indicated by the arrows.

**Figure 2:**
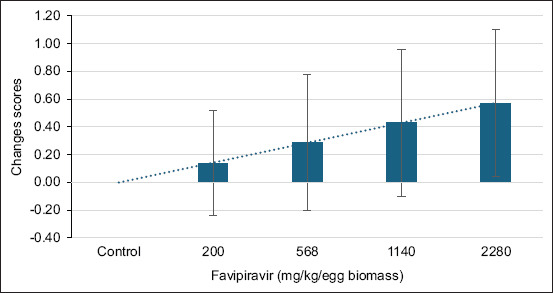
Morphological changes in embryos treated with varying doses of favipiravir. The bar graph illustrates the dose-dependent increase in morphological change scores, with error bars representing the variability (standard deviation) at each dose. The dotted trendline highlights the progressive effect of increasing doses.

Macroscopic examination revealed dose-dependent morphological abnormalities. Lower doses caused minimal changes, whereas higher doses, particularly Group E, showed pronounced hemorrhaging (angioedema) (Figure 1). The progressive increase in lesion severity was quantified in morphological change scores (Figure 2).

#### Biochemical alterations in allantoic fluid

AAF analysis showed dose-dependent changes in liver and kidney function markers (Table 3).

**Table 3:** Concentrations of biochemical parameters of allantoic fluid on day 7 after treatment with favipiravir.

Biochemical indices	A (control)	B (200 mg/kg)	C (540 mg/kg)	D (1,140 mg/kg)	E (2,280 mg/kg)
AST					
Mean ± SD	32.00 ± 7.77	35.71 ± 7.39	28.14 ± 4.91	37.14 ± 11.57	35.29 ± 7.70
95% CI (mean)	24.82–39.18	28.88–42.55	23.60–32.69	26.44–47.84	28.17–42.40
ANOVA p-value/effect size (η²)			p = 0.266		
			η^2^ = 0.155		
ALT					
Mean ± SD	35.57 ± 3.10	33.5 ± 2.37	29.29 ± 8.10	36.57 ± 9.90	34.14 ± 3.34
95% CI (mean)	32.70–38.44	31.38–35.76	21.80–36.77	27.42–45.72	31.06–37.23
ANOVA p-value/effect size (η^2^)			p = 0.244		
			η^2^ = 0.162		
ALP					
Mean ± SD	27.13 ± 3.64	34.00 ± 6.02	33.88 ± 4.61	40.88 ± 3.80	41.88 ± 2.64
95% CI (mean)	24.08–30.17	28.96–39.04	30.02–37.73	37.70–44.05	39.67–44.08
ANOVA p-value/effect size (η^2^)			p < 0.001*		
			η^2^ = 0.642		
*Post hoc* (Tukey’s HSD p-value) control versus test groups	-	p = 0.023*	p = 0.026*	p < 0.001*	p < 0.001*
Urea					
Mean ± SD	23.59 ± 2.10	44.71 ± 7.76	35.39 ± 5.77	30.17 ± 2.73	42.86 ± 2.97
95% CI (mean)	21.65–25.53	37.54–51.89	30.06–40.73	27.64–32.70	40.11–45.60
ANOVA P-value/effect size (η^2^)			p < 0.001*		
			η^2^ = 0.760		
*Post hoc* (Tukey’s HSD p-value) control versus test groups	-	p < 0.001*	p < 0.001*	p = 0.101	p < 0.001*
Creatinine					
Mean ± SD	0.94 ± 0.08	1.14 ± 0.34	1.11 ± 0.28	0.95 ± 0.08	1.14 ± 0.25
95% CI (mean)	0.87–1.02	0.83–1.46	0.85–1.37	0.88–1.02	0.91–1.37
ANOVA p-value/effect size (η^2^)			p = 0.273		
			η^2^ = 0.153		
Kruskal-Wallis p-value/effect size (ε^2^)			p = 0.410		
			ε^2^ = 0.000		

Biochemical values in the asterisk are significantly different from the control. SD = Standard deviation, CI = Confidence interval, ANOVA = Analysis of variance, ALP = Alkaline phosphatase, HSD = Honestly significant difference, AST = Aspartate aminotransferase, ALT = Alanine transaminase

- AST and ALT: No significant differences, indicating mild effects.
- ALP: Significantly elevated across all treated groups (p < 0.001, η^2^ = 0.642), reflecting a strong dose-dependent effect.
- Urea: Highly significant increase with treatment (p < 0.001, η^2^ = 0.760), with *post hoc* differences in Groups B, C, and E compared with control.
- Creatinine: No significant differences across groups (by ANOVA and Kruskal-Wallis), suggesting no treatment-related effect.

#### Histopathological changes

**Figure 3:**
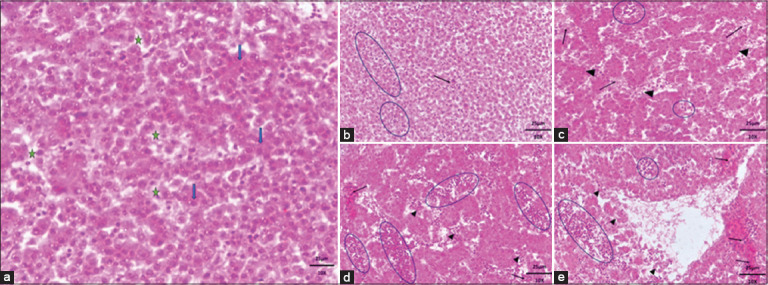
Histopathological changes in embryonic livers after exposure to various doses of favipiravir. Group (a) represents the control group, showing normal liver architecture on day 17 of incubation. Hepatocytes (blue arrows) are arranged in a lobular pattern within the parenchyma, while sinusoidal spaces (green stars) are visible as faint, irregular clear areas between hepatocytes where blood flows through the liver. Group (b) illustrates hepatocyte hydropic degeneration (arrow) and the presence of inflammatory cells with dark, round nuclei distributed diffusely among hepatocytes and within sinusoids (circles). In group (c), inflammatory cells (circles) are evident along with vacuolation in the sinusoidal region (arrowhead) and congested hepatocytes (arrow). Group (d) reveals congested and necrotic hepatocytes (arrows), extensive inflammatory cells within the sinusoids (circles), and vacuolation and necrotic areas resulting from hepatic degeneration (arrowhead). Finally, group (e) shows severe fibrinous damage in vascular areas (arrowhead), congested hepatocytes undergoing degeneration (arrow), and deformed sinusoids with congestion and inflammatory changes (circle). Hematoxylin and eosin staining; scale bar = 25 μm.

Histopathology revealed dose-dependent hepatotoxicity (Figure 3).

- Group A (control): Normal hepatocyte and sinusoidal architecture
- Group B: Mild inflammation
- Group C: Early inflammation without necrosis
- Group D: Inflammatory aggregation with necrosis
- Group E: Extensive necrosis and widespread inflammation.

Pearson’s correlation showed a strong positive association between ALP activity and liver lesion scores (r = 0.82, r^2^ = 0.68), confirming biochemical and histological agreement.

### Therapeutic efficacy of favipiravir against VVNDV

#### Embryo survival and growth

**Figure 4:**
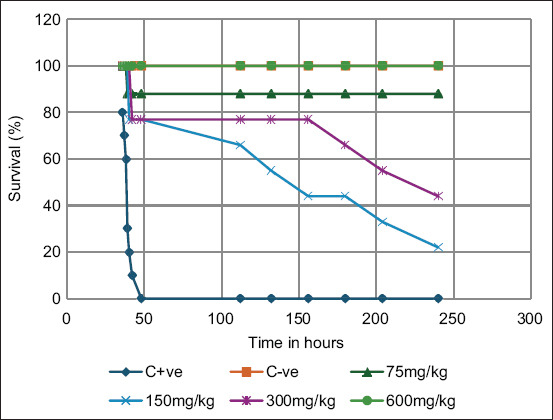
Kaplan-Meier survival curve showing the effect of different antiviral drug doses on embryo survival over time. The X-axis represents time (hours), and the Y-axis indicates the percentage of surviving embryos. The positive control group (C + ve, blue line), representing infected but untreated embryos, exhibited complete mortality within 48 h. In contrast, the negative control group (C−ve, orange line), comprising uninfected and untreated embryos, maintained 100% survival throughout the study. Doses of 75 mg/kg (gray line), 150 mg/kg (yellow line), 300 mg/kg (light blue line), and 600 mg/kg (green line) revealed a dose-dependent increase in survival rates. Notably, the 600 mg/kg dose provided complete protection, whereas the 300 mg/kg dose maintained approximately 60% survival at 250 h.

Favipiravir significantly improved survival in VVNDV-infected embryos. At 300 and 600 mg/kg (Groups G5 and G6), survival reached 100%, comparable to negative controls and significantly higher than the infected, untreated group (p < 0.05) (Figure 4).

**Figure 5:**
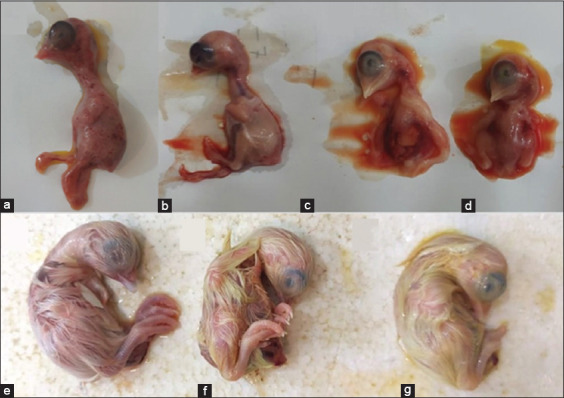
Gross morphological analysis of embryonated chicken eggs (ECEs) under different experimental conditions. Panel “a” shows the embryos in the positive control group (G1) infected with Newcastle disease virus (NDV), exhibiting extensive cutaneous hemorrhages on the 2^nd^ day after inoculation. Panels “d” and “c” represent dead embryos from groups G3 and G4, respectively, treated with favipiravir at doses of 75 and 150 mg/kg, respectively, against NDV infection. Panel “b” depicts the negative control (G2) embryo on day 3 after inoculation, appearing normal. Panels “e,” “f,” and “g” display surviving embryos from the negative control group (G2), 300 mg/kg favipiravir-treated group (G5), and 600 mg/kg favipiravir-treated group (G6), respectively, on day 7 post-inoculation.

**Figure 6:**
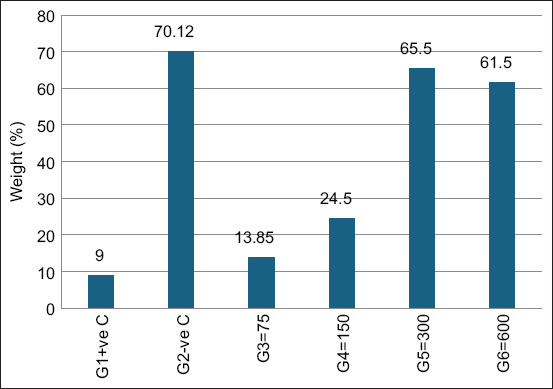
Embryo weight relative to egg weight: weight by weight percentage. (%WW) after exposure to varying doses of favipiravir against Newcastle disease virus *in ovo.*

Morphological assessment further confirmed protective effects (Figure 5). Favipiravir-treated embryos also showed significantly increased relative embryo weight compared with the positive control group (Figure 6).

#### Inhibition of viral replication

**Figure 7:**
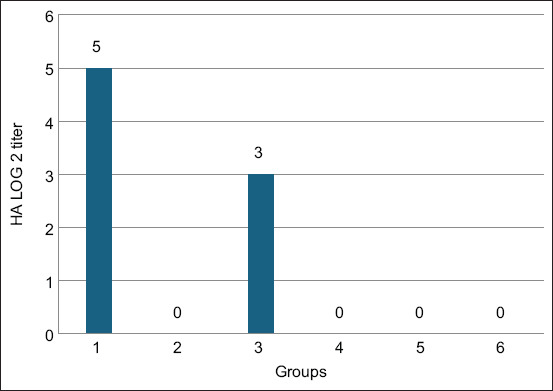
Hemagglutination titers of Newcastle disease virus in the allantoic fluid of ECEs were measured 72 h after treatment with various doses of favipiravir.

**Figure 8:**
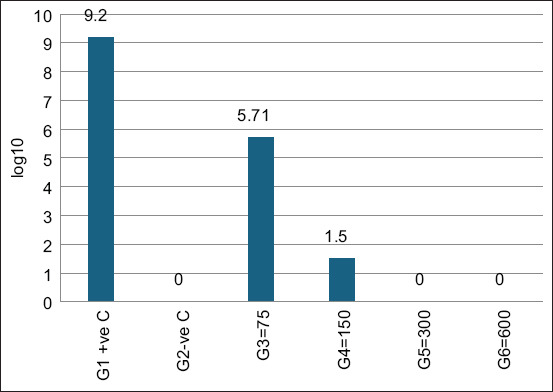
Newcastle disease virus (NDV) titers (50% egg infectious dose log10) in groups infected with NDV and treated with favipiravir.

Favipiravir effectively suppressed VVNDV replication:

- HA titers: Completely undetectable in Groups G5 and G6, compared with high titers in controls (Figure 7)
- EID_50_ titers: Undetectable in Groups G5 and G6, while controls retained high viral loads (Figure 8).

These results confirm potent antiviral efficacy at therapeutic doses.

### MDA

**Figure 9:**
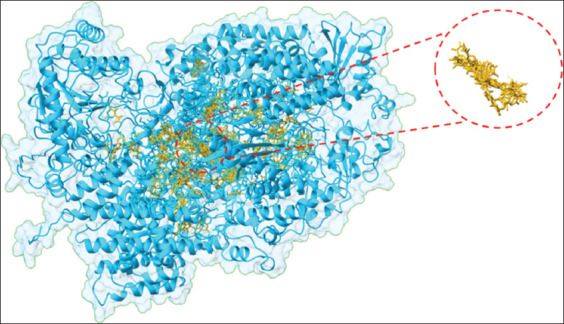
Identification of binding pockets occupied by the generated conformations of favipiravir revealed through blind docking. The region with the densest cluster and the selected pocket are highlighted.

Blind docking identified multiple druggable cavities on NDV RdRp (Figure 9). The pocket with the densest ligand conformations was selected for detailed analysis.

Key interacting residues included Asp588, Lys594, Arg1189, Val1190, Pro1191, Tyr1192, Leu1193, Gly1194, Ser1195, Lys1196, Thr1197, Gln1198, Glu1199, Arg1200, Ile1293, and Asp1446.

Favipiravir demonstrated strong binding affinity with a docking score of −4.8799. Four hydrogen bonds were identified:

- Arg1189 formed two H-bonds with the pyrazine ring (2.68 and 2.14 Å)
- Tyr1192 formed one H-bond with the ligand’s carbonyl oxygen (2.61 Å)
- Ser1288 formed one H-bond with the ligand’s amide group (2.50 Å).

**Figure 10:**
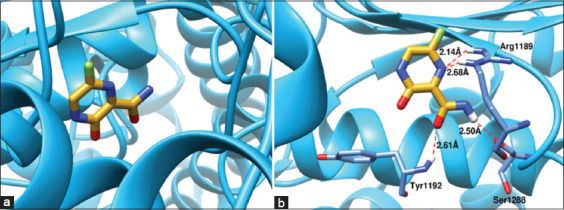
(a) Favipiravir (goldenrod sticks) orientation in the binding pocket of RNA-dependent RNA polymerase of Newcastle disease virus and (b) 3D representation of intermolecular interaction pattern. The residues involved in hydrogen bonding are displayed as cornflower blue sticks, and the hydrogen bonds are represented by dotted red lines.

These interactions (Figure 10) suggest that favipiravir directly interferes with RdRp function through stable electrostatic interactions and hydrogen bonding, explaining its observed antiviral efficacy.

## DISCUSSION

### Therapeutic significance of favipiravir

This study explored the therapeutic potential of favipiravir against VVNDV, a major threat to the poultry industry. Results confirmed that favipiravir possesses potent antiviral activity in an *in ovo* model, while also underscoring the importance of dose optimization to mitigate toxicity.

### Toxicity profile of favipiravir

The *in ovo* toxicity assessment demonstrated a clear dose-dependent relationship between favipiravir concentration and adverse outcomes on embryo survival, growth, and organ morphology. Pronounced toxicity was observed at the highest dose (2,280 mg/kg), which significantly reduced embryo survival. Doses above 1,200 mg/kg were associated with marked toxicity, whereas doses between 200 and 1,200 mg/kg produced only mild effects, emphasizing the necessity of careful dosage optimization.

The observed embryonic mortality at higher doses aligns with earlier reports describing teratogenic and embryotoxic effects of favipiravir in animal models [18, 19]. This toxicity is likely linked to the drug’s mechanism of action, whereby incorporation into viral RNA may also interfere with host cellular processes at elevated concentrations. Comparable findings have been reported for nitazoxanide, another broad-spectrum antiviral that exhibits dose- and time-dependent cytotoxicity *in vitro* [1].

Favipiravir reduced embryo growth primarily at the highest dose (2,280 mg/kg), while lower doses had minimal impact. Disruption of gut absorption and impaired embryonic development may underlie these effects [20]. Morphological abnormalities, including angioedema, observed in this study support clinical reports of favipiravir-induced angioedema in COVID-19 patients [21]. Such effects may result from mast cell degranulation or activation of the kallikrein–kinin system, potentially compromising the embryonic vascular network essential for normal development [22, 23].

Biochemical analyses confirmed hepatotoxicity at higher doses, with elevated ALP and urea levels correlating with histopathological liver damage. A strong positive correlation (r = 0.82, r^2^ = 0.68) between ALP and lesion scores suggests that ALP may serve as a reliable biochemical indicator of hepatic injury. These findings are consistent with reports of liver enzyme elevations as markers of drug-induced liver injury in other antiviral studies [24]. In addition, favipiravir-induced elevations in uric acid may reflect its metabolism to the inactive M1 metabolite by aldehyde oxidase and xanthine oxidase, with subsequent effects on renal uric acid transport organic anion transporter 1, organic anion transporter 3, and Urate transporter 1, pathways [25].

### Antiviral efficacy against VVNDV

Despite dose-related toxicity, favipiravir demonstrated remarkable antiviral efficacy at non-toxic concentrations. Complete inhibition of VVNDV replication was observed at 300 and 600 mg/kg, with outcomes including 100% embryo survival, increased weight gain, and undetectable viral titers.

Interestingly, the 75 mg/kg dose showed superior survival compared with intermediate doses (150 and 300 mg/kg). This may represent a balance between antiviral activity and physiological stress. At intermediate doses, embryos may have experienced additional metabolic burden, partially offsetting antiviral benefits. Conversely, 600 mg/kg appeared sufficient to reduce viral replication early, thereby lowering stress and improving outcomes. This pattern suggests the existence of an optimal therapeutic window.

These findings corroborate reports of favipiravir’s broad-spectrum antiviral activity against influenza [13], Ebola [26], and Lassa viruses [27]. Unlike ribavirin, favipiravir induces fewer viral RNA mutations, suggesting a favorable safety profile for virus control. Previous studies have shown only partial suppression of viral replication with other antivirals [28, 29], whereas favipiravir achieved complete clearance at effective doses.

Favipiravir-treated embryos also demonstrated improved growth and fewer lesions, counteracting the epithelial damage and malabsorption commonly induced by NDV [30]. Comparative evidence highlights favipiravir’s superior efficacy compared to other agents, such as niclosamide and fucoidan [31–33]. The embryo model itself, widely used for viral drug evaluation, provides an ideal non-immune platform to assess antiviral activity [34]. The strong efficacy observed here suggests that favipiravir could be developed further for field application in poultry, with broader potential against other RNA viruses of zoonotic concern.

### Docking insights into the mechanism of action

Molecular docking provided mechanistic confirmation of favipiravir’s activity against NDV. The drug exhibited high affinity for RdRp, with binding driven by electrostatic interactions and hydrogen bonding. Key residues (Arg1189, Tyr1192, Ser1288) were consistently involved in binding, supporting the hypothesis that favipiravir interferes directly with viral RNA synthesis.

This mechanism is consistent with its established role as a nucleoside analog, competing with natural nucleotides, causing chain termination or lethal mutagenesis [13]. Computational analysis confirmed that even subtle structural alterations in binding sites may disrupt interactions, highlighting the importance of these residues. These results improve understanding of the molecular basis of antiviral activity and provide a foundation for rational drug design of enhanced RdRp inhibitors.

### Comparison with other antiviral strategies

Currently, NDV control relies on vaccination and biosecurity, with no approved antivirals [35]. However, vaccine-resistant strains underscore the urgent need for effective therapeutic options [36]. Alternative strategies under investigation include RNA interference [11] and CRISPR-Cas-based approaches [37]. Natural and synthetic compounds, such as n-docosanol [38], tangeretin [39], neem, black seed, and curcumin [40], have also demonstrated variable activity, although their mechanisms and field applicability remain unclear.

Comparative evidence indicates that favipiravir outperforms several of these alternatives. Niclosamide primarily interferes with glycolytic pathways [29], while fucoidan targets viral HN and F proteins but achieves incomplete protection [28]. Favipiravir, already approved for human use, offers the advantage of rapid repurposing, high potency, and clear mechanistic validation, making it a strong candidate for NDV therapy.

## CONCLUSION

This study demonstrates the potent antiviral efficacy of favipiravir against VVNDV in an embryonated chicken model. Favipiravir exhibited a clear dose-dependent profile, with high doses (≥1,200 mg/kg) inducing significant embryotoxicity, hepatotoxicity, and vascular alterations, while lower non-toxic doses (75–600 mg/kg) achieved substantial viral inhibition. Notably, favipiravir at 300 and 600 mg/kg resulted in 100% embryo survival, increased embryo weight, and undetectable viral loads, underscoring its strong therapeutic potential against NDV. Docking studies further revealed high-affinity binding of favipiravir to the RdRp active site, validating its mechanism of action as a nucleoside analog and supporting its role in lethal mutagenesis of viral RNA.

The findings highlight favipiravir as a promising candidate for controlling NDV outbreaks, particularly in settings where vaccine efficacy is compromised due to emerging virulent strains. As favipiravir is already approved for human use against influenza and other RNA viruses, its repurposing in veterinary medicine offers a readily translatable option for rapid deployment during NDV outbreaks. The potential application extends beyond poultry, given the zoonotic risk of paramyxoviruses, emphasizing the drug’s One Health relevance.

Key strengths of this study include the comprehensive *in ovo* evaluation integrating survival, growth, biochemical, histopathological, and morphological outcomes; validation of antiviral activity with molecular docking insights that strengthen mechanistic understanding; and comparative evaluation demonstrating that favipiravir outperforms other tested antivirals in terms of viral clearance and embryo survival. However, certain limitations must be acknowledged, including reliance on a single viral genotype, the absence of *in vivo* trials in chickens, and the lack of long-term pharmacokinetic and safety data, which restrict the extrapolation of these results to field conditions.

Future research should therefore focus on *in vivo* validation in chickens and poultry flocks, long-term safety and pharmacovigilance, testing across diverse NDV genotypes, and the development of optimized oral or injectable formulations for mass application. The exploration of structure-activity relationships to design more selective RdRp inhibitors may further enhance therapeutic potential.

In summary, this study provides compelling evidence that favipiravir is a viable antiviral candidate against VVNDV, capable of overcoming current limitations of vaccination. By combining experimental efficacy, mechanistic validation, and translational potential, favipiravir represents a promising tool for improving NDV control strategies and reducing the global economic burden of this devastating disease.

## DATA AVAILABILITY

The data supporting the findings of this study (including raw biochemical measurements, docking outputs, and statistical analysis files) are available from the corresponding author upon reasonable request.

## AUTHORS’ CONTRIBUTIONS

NAS: Conceived and designed the study, performed all bench work, carried out statistical analysis, analyzed and interpreted the data, and drafted and revised the manuscript. ZAN: Experimental design, data analysis, and drafted the manuscript. MT: Data acquisition, histopathology, data generation, and critically reviewed the manuscript. NHK: Technical support for experiments and drafted the manuscript. MM: Performed molecular docking analysis and reviewed the manuscript. All authors have read and approved the final manuscript.
